# Anticancer effects against colorectal cancer models of chloro(triethylphosphine)gold(I) encapsulated in PLGA–PEG nanoparticles

**DOI:** 10.1007/s10534-021-00313-0

**Published:** 2021-04-27

**Authors:** Alessio Menconi, Tiziano Marzo, Lara Massai, Alessandro Pratesi, Mirko Severi, Giulia Petroni, Lorenzo Antonuzzo, Luigi Messori, Serena Pillozzi, Damiano Cirri

**Affiliations:** 1 Department of Experimental and Clinical Medicine, University of Florence, Viale G.B. Morgagni 50, 50134 Firenze, Italy; 2 Department of Pharmacy, University of Pisa, Via Bonanno Pisano 6, 56126 Pisa, Italy; 3 Laboratory of Metals in Medicine (MetMed), Department of Chemistry “U. Schiff”, University of Florence, Via della Lastruccia 3, 50019 Sesto Fiorentino, Italy; 4 Department of Chemistry and Industrial Chemistry (DCCI), University of Pisa, Via G. Moruzzi 13, 56124 Pisa, Italy; 5 Azienda Ospedaliero-Universitaria Careggi, S.C. Oncologia Medica 1, Florence, Italy; 6 DI.V.A.L Toscana S.R.L., Via Madonna del Piano, 6, 50019 Sesto Fiorentino, Italy

**Keywords:** PLGA–PEG nanoparticles, Auranofin, Anticancer complexes, Colorectal cancer

## Abstract

Chloro(triethylphosphine)gold(I), (Et_3_PAuCl hereafter), is an Auranofin (AF)-related compound showing very similar biological and pharmacological properties. Like AF, Et_3_PAuCl exhibits potent antiproliferative properties in vitro toward a variety of cancer cell lines and is a promising anticancer drug candidate. We wondered whether Et_3_PAuCl encapsulation might lead to an improved pharmacological profile also considering the likely reduction of unwanted side-reactions that are responsible for adverse effects and for drug inactivation. Et_3_PAuCl was encapsulated in biocompatible PLGA–PEG nanoparticles (NPs) and the new formulation evaluated in colorectal HCT-116 cancer cells in comparison to the free gold complex. Notably, encapsulated Et_3_PAuCl (nano-Et_3_PAuCl hereafter) mostly retains the cellular properties of the free gold complex and elicits even greater cytotoxic effects in colorectal cancer (CRC) cells, mediated by apoptosis and autophagy. Moreover, a remarkable inhibition of two crucial signaling pathways, i.e. ERK and AKT, by nano-Et_3_PAuCl, was clearly documented. The implications of these findings are discussed.

**Supplementary Information:**

The online version contains supplementary material available at 10.1007/s10534-021-00313-0.

## Introduction

Auranofin (AF) is an established gold(I) drug for the treatment of rheumatoid arthritis, in clinical use since 1986 (Bombardier et al. 1986). In the last decade, AF and in general gold-based compounds, have been extensively reconsidered as potential anticancer agents characterized by an original and DNA-independent mode of action (Pratesi et al. 2014; Micale et al. 2014; Marzo et al. 2017; Magherini et al. 2018; Scalcon et al. 2018). The first papers on AF and its anticancer activity revealed that this gold complex is an effective agent in vitro toward melanoma and leukaemia cells (Mirabelli et al. 1985); its activity toward ovarian and non-small-cell lung cancer cells was later documented (Roder and Thomson 2015; Li et al. 2016). It is now widely recognized that the pharmacologically active portion of the AF molecule is the cationic fragment [Au(PEt_3_)]^+^ (Pratesi et al. 2018; Zoppi et al. 2020) while the thiosugar moiety mainly acts as a carrier ligand improving the bioavailability and the gold complex pharmacokinetic profile when orally administered (Marzo et al. 2017). Based on these arguments, recently, we started a systematic and comparative evaluation of a series of AF-related compounds (Marzo et al. 2017, 2018; Tolbatov et al. 2020). Our attention was firstly focused on AF-related complexes where the thiosugar moiety is replaced by halide ligands. Interestingly, two of them—i.e. Et_3_PAuCl and Iodo(triethylphosphine)gold(I)-manifested a fully retained or even improved anticancer activity toward colorectal and ovarian cancer cells (Marzo et al. 2017, 2019). Since Et_3_PAuCl possesses a pharmacological profile closely resembling AF (Sutton et al. 1972), it is plausible-at least in principle- to further enhance its pharmacological actions by reducing those unwanted (“off target”) side reactions that often limit drug bioavailability/cellular distribution. Indeed, the Au centre can react with sulphur-containing solvent exposed aminoacidic residues of serum proteins (Zoppi et al. 2020).

Among several approaches, to achieve this goal, a reasonable strategy is given by the preparation of biocompatible nanostructures as smart platforms for drug delivery. Indeed, the use of biocompatible nanocarriers is widely recognized as a very promising strategy for pharmaceutical applications (Afshari et al. 2014; Johnstone et al. 2016; Anari et al. 2016; Merlino et al. 2017). Thus, we have explored whether the activity of Et_3_PAuCl toward CRC cells might be enhanced by encapsulation of this compound in fluorescent PLGA–PEG nanoparticles bearing a rhodamine fluorophore for tracking (Cheng et al. 2007; Tian et al. 2017; Poursharifi et al. 2018). A detailed and comparative characterization of the anticancer effects of these constructs has been carried out in 2D or 3D cell cultures, highlighting the promising features of this novel Et_3_PAuCl-encapsulated formulation.

## Materials and methods

All the reagents were provided by Merck-Sigma Aldrich and used without further purification. Et_3_PAuCl was purchased from Sigma-Aldrich (code: 288225). Solvents were also used without further purification.

### Preparation of polymeric matrix

**Fig. 1:**
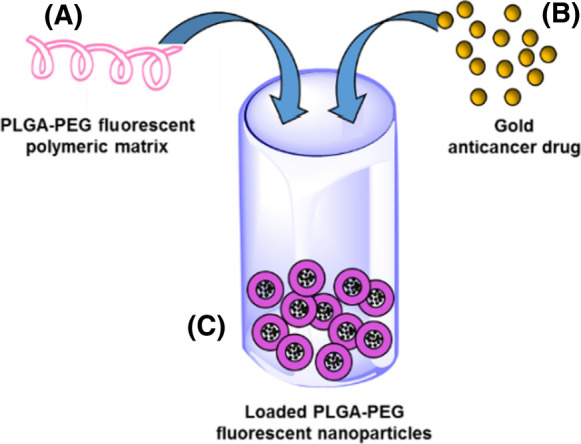
Schematic representation for the preparation of the loaded PLGA–PEG nanoparticle. **a** PLGA–PEG polymer covalently functionalized with the Rhodamine B fluorescent probe; **b** the yellow spheres represent Et_3_PAuCl; **c** the nanoparticle after gold complex loading

PLGA–C=O–NH–PEG–COOH: 251 mg of 50:50 PLGA (mw 7000–17,000 g/mol; 0.021 mmol) were solubilised in 8 mL of anhydrous dichloromethane. 14 mg of EDC·HCl (1-ethyl-3-(3-dimethylaminopropyl)carbodiimide hydrochloride) (0.073 mmol) were added to the solution under stirring. After 10 min 7 mg (0.061 mmol) of N-Hydroxysuccinimide were also added. After 1 h, 77 mg of H_2_N–PEG–COOH (3400 g/mol) and 7 µL of N,N-Diisopropylethylamine were then added. The solution was stirred at room temperature for 18 h. Next, the solvent was evaporated under reduced pressure. The crude product was solubilized with 10 mL of chloroform, then the solution was washed twice with 10 mL of brine. The organic phase was dried with MgSO_4_ and filtered. The desired product was obtained after prolonged evaporation of solvent under reduced pressure (242 mg; 0.017 mmol; 81% yield). ^1^HNMR is consistent with the data already reported in literature (Cheng et al. 2007) (see Fig. 1).

RhB–C=O–PLGA–C=O–NH–PEG–COOH: In a 50 mL flask were added 41 mg of Rhodamine B, 8 mL of anhydrous dichloromethane, 242 mg of PLGA–PEG–COOH (0.017 mmol) previously synthesized, 19 mg (0.099 mmol) of EDC·HCl and 9.5 µL (0.05 mmol) of N,N-Diisopropylethylamine. The reaction mixture was stirred at r.t. for 18 h, then the suspension was dried under reduced pressure. The crude product was solubilized with 5 mL of dichloromethane and the solution was washed with 10 mL of brine. The organic phase was dried with MgSO_4_ and filtered. The volume was reduced with rotary evaporator and 2 mL of methanol were added. The product was finally dried under reduced pressure and 191 mg of rhodaminated PLGA were obtained (0.013 mmol; yield 76%).

^1^HNMR (400.13 MHz; CDCl_3_): 7.98 (d; 7.56 Hz; RhB moiety); 7.62 (dt; RhB moiety); 7.56 (dt; RhB moiety); 7.20 (d; 7.50 Hz; RhB moiety); 6.54 (m: RhB moiety); 6.43 (m; RhB moiety); 6.32 (m; RhB moiety); 5.20 (b; CH lactic moiety); 4.81 (m; CH_2_ glycolic moiety); 3.64 (s; PEG moiety); 3.5 (q; 7.07 Hz; RhB); 1.57 (m; CH_3_ lactic moiety); 1.16 (t; 7.05 Hz; RhB) (Fig. S1).

### Nanoparticles preparation

The particles were precipitated by solubilization of the polymeric matrix in acetonitrile at the concentration of 5 mg/mL in presence of 1% (weight) of Et_3_PAuCl by dropwise addition of the solution to 4×  volume of stirring water. 2.24 mg of Et_3_PAuCl were solubilized in 4.48 mL of acetonitrile. 1 mL of the obtained solution (1.43 mM) was diluted to 10 mL with acetonitrile to a final concentration of 0.143 mM. Afterward, 1 mL of this latter solution was used to solubilize 5.8 mg of polymeric matrix. The obtained solution was added dropwise into 4 mL of stirring water and the organic solvent removed by evaporation under reduced pressure for 40 min. After the evaporation process, 3.6 mL of an aqueous suspension of Et_3_PAuCl loaded nanoparticles were obtained. The final concentration of Et_3_PAuCl, determined through ICP-AES analysis, was 0.04 mM (Cheng et al. 2007; Park et al. 2008; Tian et al. 2017; Poursharifi et al. 2018).

### Spectroscopic properties

Absorption proprieties were investigated using an UV–Vis CaryBio50 spectrophotometer. A suspension of 246 µg/mL was investigated for determining the λ of maximum absorbance (562 nm). To further asses the complete binding of the rhodamine dye to the polymeric structure, UV–Vis analysis was repeated on the nanoparticle solution (at the above concentration) after centrifugation using 100 kDa nominal cut-off Amicon filter. To this end 5 mL of solution were centrifuged for 2 min at 4428 RCF. Next, the filtered solution was investigated trough absorbance experiments revealing only tiny traces of unbounded rhodamine (Fig. S2A). For a better quantification of unbounded rhodamine, the previous prepared samples were analyzed through luminescence emission experiments using a Perkin Elmer LS55 fluorescence spectrometer. Analysis were performed with an excitation wavelength of 530 nm (Fig. S2B). Through a comparison between the heights of fluorescence emission spectra, we deduced that fluorescence emission due to unbounded dye was about the 6% of the total. The λ of maximum fluorescence emission turned out to be 582 nm.

### Et_3_PAuCl retention

The retention of the loaded Et_3_PAuCl from nanoparticles was evaluated through ICP-AES spectroscopy. First of all, loaded nanoparticles were prepared as reported above. Then, after 24 h from the formation of nanoparticles, 5 mL of suspension were centrifuged in a 100 kDa cut-off Amicon filter. The filtered solution was analyzed for determining the residual gold amount in comparison with initial addition of gold complex. The value of gold retention, obtained as a mean of analysis performed on three different batches, turned out to be 92.7% (Table S2).

### Gold complex solutions

For cellular experiments, Et_3_PAuCl was dissolved in DMSO at a concentration of 10 mM being the complex very stable in this solvent (Marzo et al. 2017). The stock solution was stored at − 20 °C. The following solutions were prepared through accurate dilutions by adding RPMI medium. Instead, the nanoparticle solutions were dissolved in ultrapure water at a concentration of 40 µM. The stock solution was stored at 4 °C.

### ICP-AES experiments (uptake)

The Au concentration in cells was measured by a Varian 720-ES Inductively Coupled Plasma Atomic Emission Spectrometer (ICP-AES) equipped with a CETAC U5000 AT+ ultrasonic nebulizer, this latter allowing to increase the method sensitivity. Cellular pellets were digested in a thermo-reactor at 80 °C for 6 h with 6 mL of 30% HNO_3_. Next, 5.0 mL of each sample were spiked with 1 ppm of Ge used as an internal standard and analysed. Calibration standards were prepared by gravimetric serial dilution from a commercial standard solution of Au at 1000 mg/L. The wavelength used for Au determination was 267.594 nm whereas for Ge the line at 209.426 nm was used. The operating conditions were optimized to obtain maximum signal intensity, and between each sample, a rinse solution of HNO_3_ suprapure grade was used in order to avoid any “memory effect”.

### Dynamic light scattering experiments (DLS)

Once prepared, the size of NPs was determined on the obtained suspension. To this end, Dynamic Light Scattering measurements were carried out on a Brookhaven BI 9000AT apparatus equipped with a Nd:YAG laser, Coherent Innova, λ = 532 nm.

### Cell culture

Human colorectal cancer (CRC) cell line HCT-116 was cultured in RPMI-1640 medium, and HEK 293 in DMEM (Euroclone; Milan, Italy), both medium supplemented with 10% fetal bovine serum (FBS). The cell lines were cultured at 37 °C in humidified atmosphere and 5% CO_2_.

### Cell viability assay

#### Trypan blue assay

The 50% inhibitory concentration (IC_50_) of the tested compounds were determined by cell viability assay, through the Trypan blue exclusion test (Sigma-Aldrich). Cells were seeded in 96-well plates (Costar Corning) at 1 × 10^4^ cells/well in RPMI complete medium. The cells were incubated for 24 h before adding the compounds at 0, 50, 75, 100, 150, 175, 200, 250, 275 and 400 nM; after the administration of the compounds, cells were further incubated for 24 h. Then, cells were harvested, and alive cells were counted using a hemocytometer. Data were mean values of the viable cell percentage of three independent experiments. The IC_50_ values were obtained by averaging the experiments and fitting with Hill1-type equation of Origin Software (Microcal Origin 8.0 software; Origin Lab Corporation, Northampton, MA).

#### MTT assay

Briefly, HCT116 were seeded into 96-well plates in a final concentration of 2000 cells/well and incubated for 24 h at 37 °C. To test the toxicity, the cells in each well were treated with the compounds at 0, 50, 100 and 200 nM. After 24 h of incubation, cell viability was determined: 80 µL of MTT-medium was added to each well for 4 h. Then, the MTT solution was discard and 120 µL of a 2-propanol/HCl solution was added to dissolve the formazan crystals and the absorbance was measured at 490 nm.

### Morphology analysis

HCT-116 cells were seeded in 24-well plates at 4 × 10^4^ cells/well in complete medium and incubated for 24 h. Cells, treated for 24 h as well as before (0, 100, 200 nM), were then harvested and their morphology was studied. Every condition was fixed on two spots of a histological slide using a refrigerated centrifuge to 728 RCF for 5 min. Afterwards, they were overnight dried at room temperature and stained with May-Grunwald Giemsa. Finally, we took images (Microscope Nikon Eclipse E200 with lens 40× ) and analyzed the effects in term of morphology alterations, using ImageJ software.

### Cell cycle analysis

The effect of Et_3_PAuCl and its encapsulated formulation were also measured on cell cycle distribution with flow cytometry, using a staining solution with propidium iodide [50 µg/mL propidium iodide, 0.1% [w/v] trisodium citrate, 0.1% NP40 (or triton x-100)]. HCT-116 cells were seeded in 24-well plates at a cell density of 3 × 10^4^ cells per well, to which gold complexes were added at: 0, 100, 200 nM. Then, cells were incubated for 24 h. At the end of incubation, cells were harvested and incubated in the staining solution for 30 min in the dark at 4 °C. The DNA content of cells was assessed using a BD FACSCanto (Becton Dickinson, Franklin Lakes, NJ, USA) and the percentage of cells in each cell cycle phase was determined by the ModFit LT 3.0 analysis software.

### 2D Proliferation assay

HCT-116 cells were cultured in 96-well plates and it was seeded at a cell density of 1 × 10^4^ per well in RPMI medium. After 24 h of incubation, cells were treated from 0 to 200 nM and they were further incubated for 24 h, 48 h and 72 h. Viable cells were determined using the Trypan Blue exclusion as described above. All experiments were performed in triplicate for each data point.

### Western blot analysis

Proteins were separated by 7.5% gel and electroblotted on PVDF membranes, following a general protocol. The membranes were blocked in 5% BSA in TPBS for 3 h at room temperature, incubated overnight in the primary antibody solutions at 4 °C, incubated in the corresponding peroxidase-conjugated secondary antibody solution for 1 h at room temperature and added ECL to achieve the chemiluminescent signals. The following primary antibodies were used for WBs, at the indicated concentrations. The rabbit pAb against phospho-44/42 MAPK (Erk1/2) (Thr202/Tyr204, #9101; dilution 1:1000) were purchased from Cell Signaling Technology. The mouse mAb against phospho-AKT1/2/3 (clone B-5, sc-271966; dilution 1:500), the rabbit pAb against AKT1/2/3 (clone H-136, sc8312; dilution 1:500), and the rabbit pAb against ERK1/2 (clone H-72, sc-292838; dilution 1:200) were purchased from Santa Cruz Biotechnology. Anti-rabbit IgG peroxidase conjugated (A0545; dilution 1:10,000) and anti-mouse IgG peroxidase antibodies (A4416; dilution 1:5000) were used as secondary antibodies.

### 3D Proliferation assay

Multicellular tumor spheroids were obtained by HCT-116 cells seeding the cells on 96-wells plate after coating the wells with a solution of sterile agarose 1.5%. After coating, cells were added at 1 × 10^3^ cells per well in complete medium. The plate was placed in the incubator at 37 °C and 5% CO_2_ for 72 h to allow the formation of three-dimensional (3D) spheroids. Next, we treated the cells with the two compounds at 0, 200 nM and we registered digital images of every spheroid at different time, starting from 0 to 144 h (0, 24, 48, 72, 96, 120, 144 h). We obtained images using a camera equipped with the microscope, 10× magnification. Every image exhibited a spheroid, whose volume could be used as a measure of the efficacy for our in vitro cancer drug analysis. Spheroid volume, determined based on the major and minor axial length of the spheroids (V = 0.5 × Length × Width^2^), were automatically calculated by SpheroidSizer1_0, a MATLAB-based and open-source software (MATLAB 2015a, MathWorks Inc.). Finally, the relative volumes were determined relating every volume to its value at time 0. The relative volumes allowed to evaluate spheroid proliferations (Chen et al. 2014).

### Fluorescence analysis

HCT-116 cells were seeded at 3 × 10^4^ on cell culture dishes 35 mm in RPMI medium and incubated 24 h. Afterwards, cells were treated at 0 and 200 nM with only Et_3_PAuCl encapsulated and they were harvested at 1 h, 2 h, 4 h after the administration. They were then fixed with formalin solution (formalin 10% in PBS) for 10 min and they were resuspended in PBS. Every cell culture dish was stored at 4 °C. The sequestration of nanoparticles by HCT-116 was quantified in term of fluorescence, thanks to the fluorescent Rhodamine B covalently linked to PLGA-PEG nanoparticles using a fluorescence microscopy [Nikon Eclipse TE300 microscope (Nikon Instruments Inc.), equipped with a Photometrics CoolSNAP CF camera (Teledyne Photometrics, Tucson AZ)]. Brightfield and fluorescence images were both collected and analyzed measuring cell fluorescence using ImageJ software. We calculated the corrected total cell fluorescence (CTCF) using the values of integrated density (IntDen hereafter) and performing the following calculation: CTCF = IntDen − (Area of selected cell × Mean fluorescence of background readings).

## Results and discussion

The loaded nanoparticles were prepared following the already established procedures (Cheng et al. 2007; Park et al. 2008; Tian et al. 2017; Poursharifi et al. 2018) as described in the experimental section. These particles are composed of a hydrophobic PLGA core surrounded by a hydrophilic PEG layer acting both as a solubilizing and a capping agent. A quite homogeneous monodispersed preparation of PLGA–PEG nanoparticles was obtained with an average size (derived from the hydrodynamic radius) of 65 nm (Table S1 supporting material). Noteworthy, the protocol used for the encapsulation process is rapid and nearly quantitative. The average content of gold inside nanoparticles was determined through ICP-AES spectroscopy; we found that 92.7% of total added gold (after 24 h) was incorporated inside nanoparticles indicating the high stability of the loaded nanoparticles and making them well suitable for the biological testing (see supporting information Table S1 and experimental section).

Nano-Et_3_PAuCl was assessed comparatively with the free gold complex for the biological effects in HCT-116 2D and 3D cell models and the distribution evaluated through fluorescence experiments (Chen et al. 2014).

**Fig. 2:**
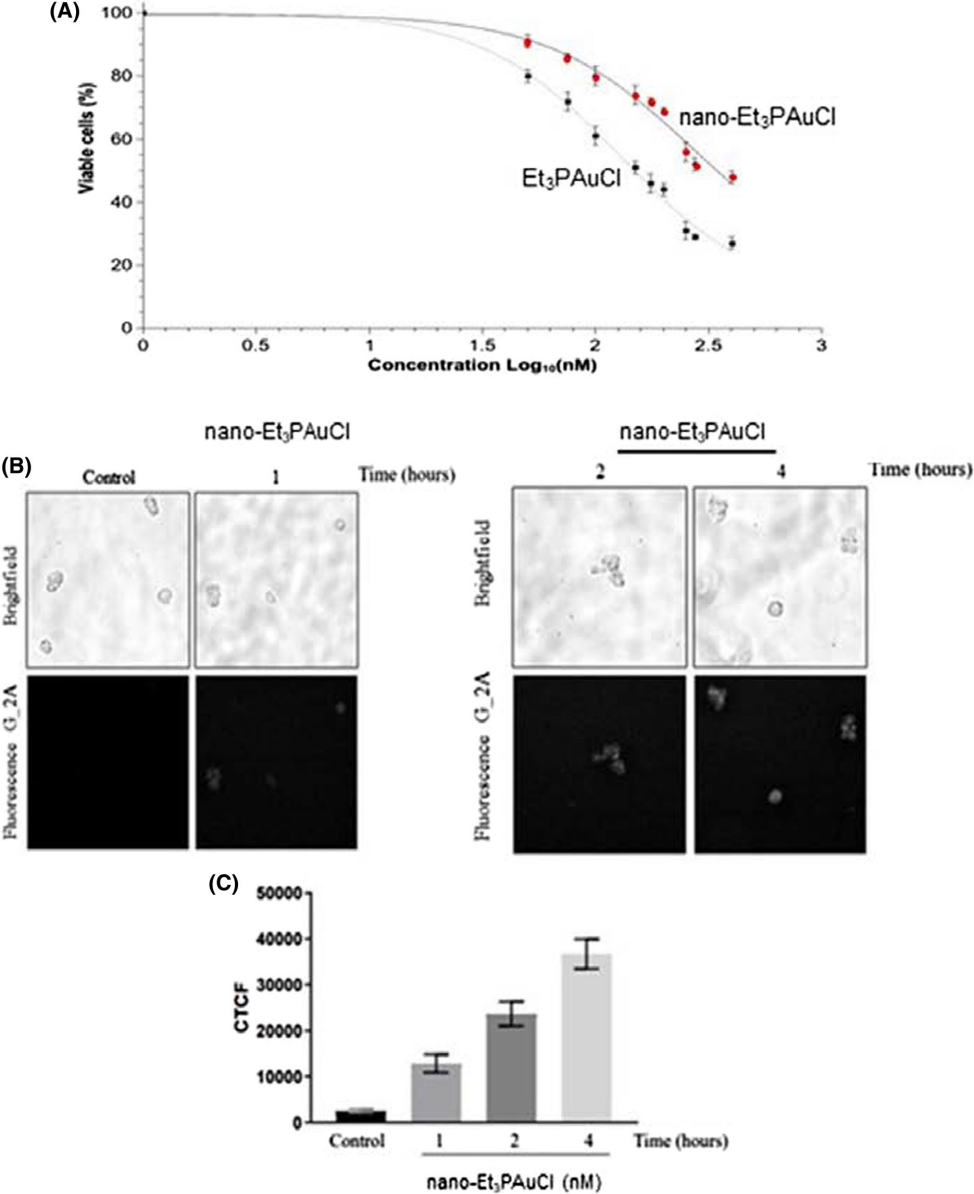
Nano-Et_3_PAuCl affects cell viability of human CRC cell line. **a** Curves and IC_50_ values (nM) of Et_3_PAuCl and of its encapsulated form in HCT-116 cell line. Cell viability was measured after 24 h of treatment, by the Trypan Blue exclusion test. Curves and IC_50_ values resulted by means of six independent experiments and they were then calculated fitting the experimental data with Origin Software Hill1-type equation. **b** Representative bright field and fluorescence images of nanoparticles cellular distribution in cancer cell suspensions, using fluorescence microscopy. **c** Histogram of fluorescence analysis relative to corrected total cell fluorescence (CTCF) obtained by ImageJ software. Data are reported as means ± SEM of 5–7 cells for each time point and every experimental condition was investigated in duplicate. The significance of differences between groups was calculated through *t* test. ^*^p < 0.05; ^**^p < 0.01. [‘CTCF’ (ctrl *vs* 1 h, p = 3.3 × 10^–5^; ctrl *vs* 2 h, p = 9.3 × 10^–8^; ctrl *vs* 4 h, p = 9.3 × 10^–10^)]

Firstly, we determined the effects of nano-Et_3_PAuCl in comparison to free Et_3_PAuCl on the viability of human CRC cells (HCT-116) after exposure to increasing concentrations of these compounds within the 0–400 nM range. After 24 h of treatment, IC_50_ values were determined (calculated through the Trypan blue exclusion test) and both nano-Et_3_PAuCl and Et_3_PAuCl were able to reduce the viability of HCT-116 cells, in a dose-dependent manner, with nano-Et_3_PAuCl being less effective (Fig. 2a).

In particular, the encapsulated form was less cytotoxic (IC_50_ = 274 nM) than Et_3_PAuCl (IC_50_ = 129 nM) (Fig. 2a). In addition, we investigated Et_3_PAuCl and its nanoformulation on HCT-116 cell viability evaluated by MTT assay and the results were virtually identical (Fig. S3).

Despite that, we could observe that both compounds reduced drastically CRC cells viability compared to untreated cells, confirming for Et_3_PAuCl the previous data published by our group (Marzo et al. 2017) (control *vs* Et_3_PAuCl 50 nM, p = 0.035; control *vs* Et_3_PAuCl 100 nM, p = 0.008; control *vs* Et_3_PAuCl 200 nM, p = 0.014; control *vs* nano-Et_3_PAuCl 100 nM, p = 0.049; control *vs* nano-Et_3_PAuCl 200 nM, p = 0.008). On the other hand, the cytotoxic active species, i.e. the free gold complex and its encapsulated formulation, when used at the same concentrations, barely affected the viability of normal human embryonic kidney cells (HEK 293), with IC_50_ values greater than 1000 nM as assessed by the same assay (data not shown).

Next, the nanoparticles cellular distribution was monitored by fluorescence microscopy being the PLGA–PEG nanoparticles covalently linked with fluorescent Rhodamine B. CRC cells (HCT-116) were treated for 1 h, 2 h and 4 h with nano-Et_3_PAuCl (200 nM) and analysed under the fluorescence microscope at different time points and the corrected total cell fluorescence (CTCF) determined. We registered the presence of the NPs inside CRC cells already after 1 h with a CTCF value of 12,825 ± 2008; notably such value increases rapidly and linearly with the longer incubation times (1 h 12,825 ± 2008 *vs* 2 h 23,702 ± 2634, p = 0.004; 1 h 12,825 ± 2008 *vs* 4 h 36,741 ± 3245, p = 5.78 × 10^–6^) (Fig. 2b and c).

As independent confirmation of the ability of the loaded NPs to enter cells, we carried out ICP-AES experiments (Table 1) using an already established protocol developed in our laboratory (Cirri et al. 2017; Pillozzi et al. 2018).

**Table 1:** Gold level (per cell) measured after exposure (3 and 24 h) of HCT116 cells to 200 nM of nano-Et_3_PAuCl

Time (h)	Concentration (nM)	Au (µg/cell)
3	200	1.7522 × 10^–9^
24	200	1.6378 × 10^–8^

**Fig. 3:**
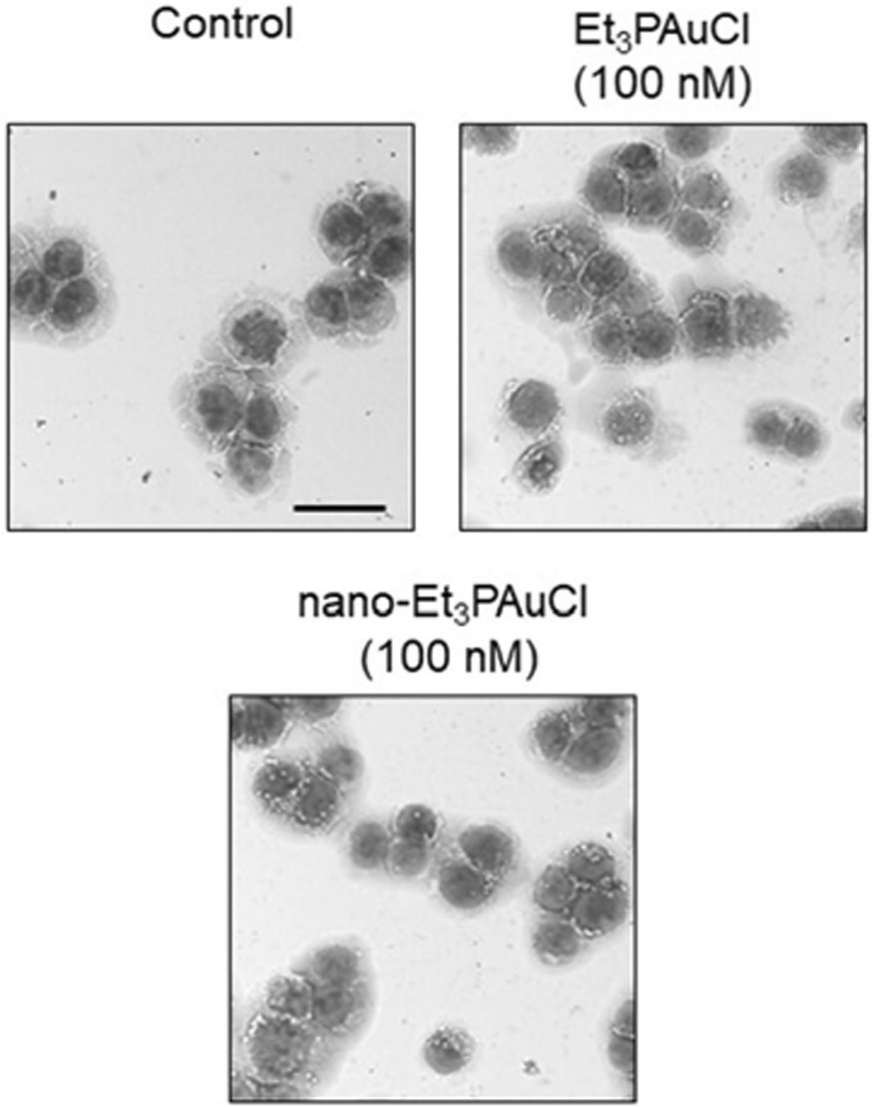
Et_3_PAuCl and its encapsulated form affect morphology of human CRC cell line. Representative images of data shown in Table 1. Scale bar = 50 µm

The ICP results nicely support and the evidence already obtained with the fluorescence approach. In fact, we confirmed as NPs are already internalized after short incubation times (3 h). Analogously, the metal uptake increases of about one order of magnitude after 24 h. In order to investigate in depth the cellular effects of the new formulation, we performed a morphological evaluation of CRC cells after 24 h of treatment: HCT-116 cells were treated with increasing concentrations of the free gold complex and its encapsulated form (0, 100 nM and 200 nM) and stained with May-Grunwald Giemsa (Fig. 3, Table 2).

**Table 2:** Morphology alterations of the compounds, given at a comparative concentration value (100 nM) on HCT-116 cells, after 24 h of treatment

Sample	Cells diameter (μm)	Nucleus/cytoplasm ratio	Vacuoles per cell
Control	26.03 ± 0.18	1.17 ± 0.09	5.21 ± 0.50
Et_3_PAuCl (100 nM)	24.57 ± 0.28^**^	1.05 ± 0.08	9.62 ± 0.79^**^
Nano-Et_3_PAuCl (100 nM)	22.85 ± 0.46^**^	1.29 ± 0.08	9.52 ± 1.96^**^

The effects were observed through May-Grunwald Giemsa staining and analyzed with ImageJ software. The analysis concerned 1012 cells per each condition, in triplicate, in term of: diameter, nucleus/cytoplasm ratio and vacuoles per cell. The significance of differences between groups was calculated through *t* test. ^*^p < 0.05; ^**^p < 0.01. [Diameter (ctrl *vs* Et_3_PAuCl 100 nM, p = 0.0009; ctrl *vs* nano-Et_3_PAuCl 100 nM, p = 2.6 × 10^–5^); vacuoles/cells (ctrl *vs* Et_3_PAuCl 100 nM, p = 0.0005; ctrl *vs* nano-Et_3_PAuCl 100 nM, p = 0.0016)].

Then, we analysed the dimensions of the treated cells in comparison to untreated cells: cells treated with Et_3_PAuCl (100 nM) showed a significant cell size reduction, calculated as the mean cell diameter with respect to control cells (control: 26.03 μm ± 0.18 *vs* Et_3_PAuCl: 24.57 μm ± 0.28, p = 0.0009), a key morphological feature of apoptosis, suggesting that the free compound does exert a pro-apoptotic effect on HCT-116 cells. Such reduction was more relevant in cells treated with nano-AFCl (at 100 nM) (control: 26.03 μm ± 0.18 *vs* nano-Et_3_PAuCl: 22.85 μm ± 0.46, p = 2.6 × 10^–5^) that induces also an increase of nucleus/cytoplasm ratio (control: 1.17 ± 0.09 *vs* nano-Et_3_PAuCl: 1.29 ± 0.08; p = 0.086) (Table 1). As expected, more pronounced effects were registered with 200 nM of Et_3_PAuCl or nano-Et_3_PAuCl treatment (data not shown).

Autophagy plays important roles in cell survival as well as in the regulation of cell death. To investigate whether the activation of autophagy contributes to the anti-proliferative effect of nano-Et_3_PAuCl, the presence of vacuoles in CRC cells was examined. HCT-116 cells were treated for 24 h with Et_3_PAuCl or nano-Et_3_PAuCl (100 nM) and the number of vacuoles per cell was measured. Although HCT-116 cells showed a conspicuous basal level of vacuoles, the number of vacuoles per cell was almost doubled after treatment with Et_3_PAuCl or with its encapsulated form suggesting the occurrence of a possible autophagic process, too (control: 5.21 ± 0.50 *vs* Et_3_PAuCl 9.62 ± 0.79 p = 0.0005; control: 5.21 ± 0.50 *vs* nano-Et_3_PAuCl 9.52 ± 1.96, p = 0.0016) (Table 2).

At the same time, we registered an increase also in terms of the percentage of CRC cells with vacuoles, that significantly increases after the treatment with both Et_3_PAuCl or nano-Et_3_PAuCl (control: 23% ± 0.7 *vs* Et_3_PAuCl 34% ± 3.0, p = 0.07; control: 23% ± 0.7 *vs* nano-Et_3_PAuCl 33% ± 3.5, p = 0.10). Conversely, no increase of the size of vacuoles was registered. Such effects are still more evident in CRC cells treated with 200 nM of the two compounds (data not shown); at this latter concentration, an increase of vacuoles dimension also emerged.

**Fig. 4:**
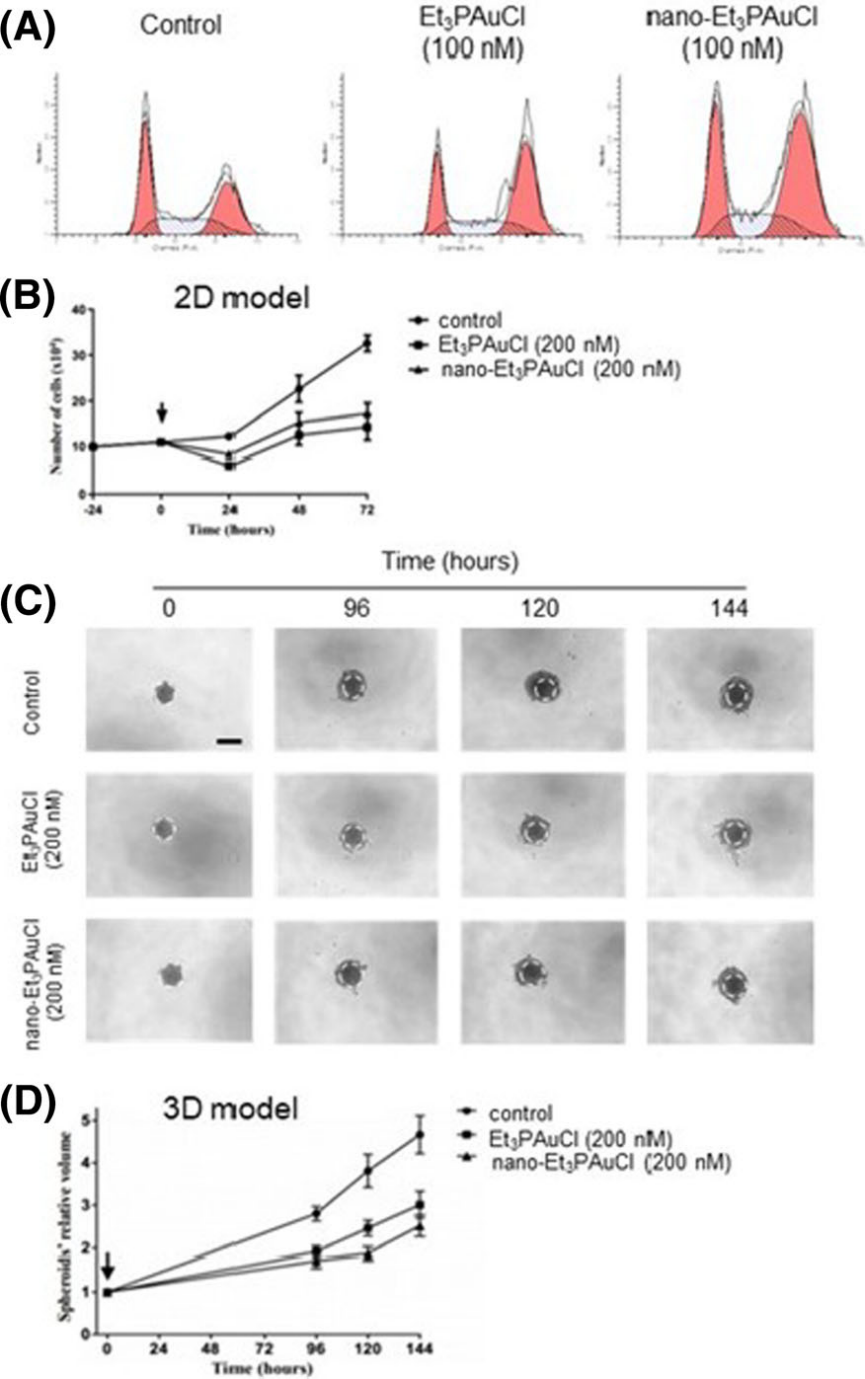
Analysis of the Et_3_PAuCl and its encapsulated form on cell cycle distribution and their effects in 2D and 3D cell cultures. **a**: Representative histograms of data shown on Table 2. **b** Effects on HCT-116 cells proliferation after incubation with 200 nM Et_3_PAuCl and its encapsulated formulation for 24, 48 and 72 h. The determination of viable cell was calculated using Trypan Blue exclusion test. Results report average value of three independent experiments ± SEM. The significance of differences between groups was calculated through *t* test. ^*^p < 0.05; ^**^p < 0.01. (Ctrl *vs* Et_3_PAuCl 24 h, p = 0.0011; ctrl *vs* nano-Et_3_PAuCl 24 h, p = 0.0058; ctrl *vs* Et_3_PAuCl 48 h, p = 0.047; ctrl *vs* Et_3_PAuCl 72 h, p = 0.0053; ctrl *vs* nano-Et_3_PAuCl 72 h, p = 0.0066). **c** Typical original spheroid images of various time conditions. Scale bar = 100 µm. **d** Histogram pertinent to relative volume of HCT116 spheroids exposed to Et_3_PAuCl and its encapsulated form (0 and 200 nM) for 0, 96, 120, 144 h. This study used the high-throughput image analysis software SpheroidSizer. Data are given by three independent experiments ± SEM. Spheroid volumes were investigated by Matlab as reported in the “Methods” section. The significance of differences between groups was calculated through *t* test. ^*^p < 0.05; ^**^p < 0.01. (Ctrl *vs* Et_3_PAuCl 96 h, p = 0.0012; ctrl *vs* nano-Et_3_PAuCl 96 h, p = 0.0009; ctrl *vs* Et_3_PAuCl 120 h, p = 0.0083; ctrl *vs* nano-Et_3_PAuCl 120 h, p = 0.0026; ctrl *vs* Et_3_PAuCl 144 h, p = 0.0112; ctrl *vs* nano-Et_3_PAuCl 144 h, p = 0.0023)

Also, we investigated in detail the effects of Et_3_PAuCl and of its encapsulated form on HCT-116 cell cycle distribution, studying the percentage of cells on the different phases of the cell cycle by propidium iodide staining. HCT-116 cells treated for 24 h with Et_3_PAuCl or nano-Et_3_PAuCl showed an enhanced percentage of cells in the G2/M phase associated to a reduced percentage of cells in S phase; this effect seems more evident in cells treated with nano-Et_3_PAuCl but is not statistically significant (percentage of cells in S phase control: 30.59 ± 5.10 *vs* Et_3_PAuCl 24.97 ± 1.61, p = 0.40; control: 30.59 ± 5.10 *vs* nano-Et_3_PAuCl 21.81 ± 2.14, p = 0.22) (Table 3 and Fig. 4a).

**Table 3:** Study of cell cycle distribution of HCT-116 cells, after 24 h of treatment with Et_3_PAuCl and its encapsulated form

Cell cycle phase	Control	Et_3_PAuCl (100 nM)	Nano-Et_3_PAuCl (100 nM)
G_0_/G_1_	31.18 ± 5.02	31.24 ± 9.11	29.58 ± 7.61
S	30.59 ± 5.10	24.97 ± 1.61	21.81 ± 2.14
G_2_/M	38.23 ± 8.62	43.79 ± 10.70	48.60 ± 9.74

Data are reported as percentage of cells on each phase of the cell cycle and as means ± SEM of four independent experiments

Thus, we observed the induction of an evident G2/M arrest, indicating that, in both cases, block the cell cycle in CRC cells impairing HCT-116 cell proliferation. Based on these premises, we studied the effects of long–term exposure of HCT-116 cells to Et_3_PAuCl or nano-Et_3_PAuCl in a 2D cell proliferation assay. 24 h after seeding HCT-116 the free gold complex or nano-Et_3_PAuCl were added at different concentrations (0, 100, 200 nM) and the number of live cells was assessed at different times (24 h, 48 h and 72 h). Data collected showed that nano-Et_3_PAuCl maintained appreciable cytotoxic properties; therefore, the combination with PLGA-PEG nanoparticles preserved the antiproliferative activity of chloro(triethylphosphine)gold(I) (Fig. 4b). To gain further insight on the anti-proliferative activity on CRC cells, the effects of Et_3_PAuCl and its encapsulated formulation, were confirmed in HCT-116 cells cultured in a three-dimensional (3D) model. The development of 3D in vitro tumor models more accurately represents human solid tumors growth. Thus, we assembled 3D tumor spheroids from HCT-116 cell line, and we exposed them to both Et_3_PAuCl and nano-Et_3_PAuCl at 200 nM. At increasing incubation times, the effects appeared clear in terms of spheroid relative volumes. At t = 96 h, t = 120 h, t = 144 h, both Et_3_PAuCl and nano-Et_3_PAuCl showed a statistically significant tumor growth inhibition compared to control samples (see figure legends for detailed values). In particular, nano-Et_3_PAuCl was more effective than Et_3_PAuCl at longer time points (t = 120 h: Et_3_PAuCl 2.46 ± 0.18 *vs* nano-Et_3_PAuCl 1.88 ± 0.15, p = 0.047) (Fig. 4c, d). These data proved that the free compound and its encapsulated form kept their effects on HCT-116 in vitro both in monolayer and in 3D tumor cell culture assays; furthermore, the encapsulation likely enhanced gold complex availability inside the 3D structures.

**Fig. 5:**
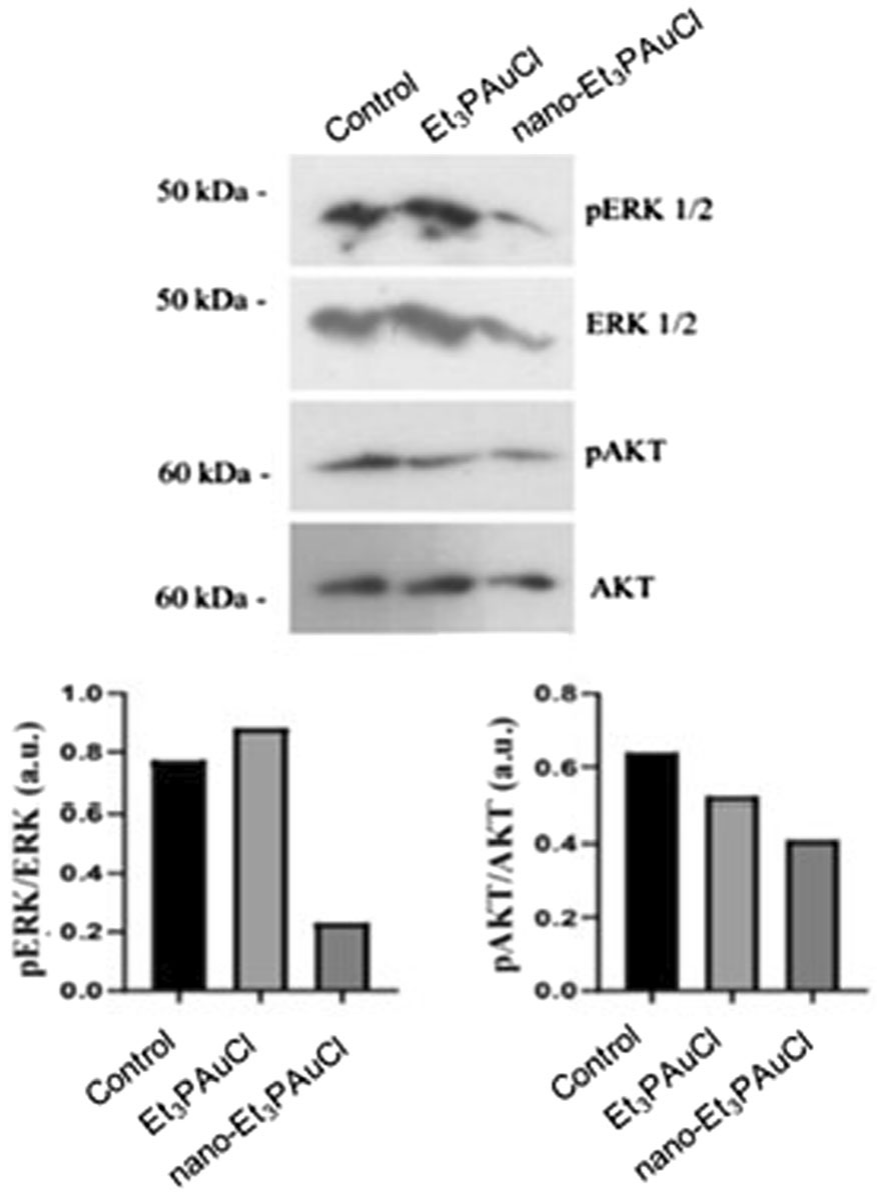
Nano-Et_3_PAuCl antitumoral activity is ERK and AKT mediated. Expression of ERK and AKT in HCT116 cells after a single treatment with Et_3_PAuCl and nano-Et_3_PAuCl at 200 nM, for 24 h. The corresponding densitometric data are reported on the bottom

At this point, since AF has been confirmed to exert significant antitumor activities (Marzo et al. 2017), we decided to explore if the study compounds do affect some relevant signaling pathways, such as AKT and ERK, whose kinases take part in the regulation of cell proliferation, cell growth and survival. The results showed that both Et_3_PAuCl and nano-Et_3_PAuCl induced, after 24 h of treatment, a remarkable downregulation of phosphorylation of AKT; in addition, nano-Et_3_PAuCl, but not Et_3_PAuCl, was also found to affect the phosphorylation levels of ERK (Fig. 5).

This difference could be induced from the increased stability of the nanoformulation, in fact our data showed that, after 24 h of observation, more than 90% of the compound was retained. Moreover, encapsulation process might lead to an improved pharmacological profile, most probably due to a higher lipophilicity of nano-Et_3_PAuCl tanks to the effect of internalization into PLGA–PEG NPs.

## Conclusions

Nowadays, the gold(I) complex AF is the leading experimental anticancer agent of the family of gold-based drugs and is currently undergoing a few clinical trials (Roder and Thomson 2015). In turn, this accession of AF to clinical trials has triggered new attention on a few AF-related complexespreviously prepared and characterized. Here, we have focused our attention on Et_3_PAuCl (Marzo et al. 2017, 2018), a compound where the thiosugar ligand of AF is replaced by a chloride ligand. Specifically, we wondered whether a nanoformulation of Et_3_PAuCl, in particular its encapsulation into PLGA–PEG nanoparticles, might result into improved pharmacological and anticancer performances. Notably, Et_3_PAuCl and other AF-related complexesbearing different substituents had been previously reported to produce important anticancer actions in vitro and in vivo on a variety of cancer cell lines (Marzo et al. 2017, 2019). Accordingly, we proceeded with the preparation of a nanoformulation of Et_3_PAuCl: the well-known PLGA-PEG nanoparticles were chosen for the encapsulation process. The size of the nanoparticles was set around a diameter of 68 nm. The above nanoformulation turned out to be stable in a physiological medium: in fact, just a minor percentage of Et_3_PAuCl is released after 24 h of observation with more than 90% of the compound retained. We performed a number of biological tests on the above Et_3_PAuCl loaded nanoparticles (nano-Et_3_PAuCl) in comparison to free Et_3_PAuCl in a HCT-116 CRC cell line. At first, we wanted to establish whether nanoformulated Et_3_PAuCl retains the cytotoxic properties of free Et_3_PAuCl. Experiments carried out in 2D and 3D CRC cell models highlighted that anti-proliferative properties were retained; in particular, nano-Et_3_PAuCl was more effective than Et_3_PAuCl at longer time points against the 3D cancer model likely due to a more favorable kinetic of release of the pharmacologically active fragment [Au(PEt_3_)]^+^. This latter aspect is of particular importance because, this augmented anticancer effects, is exerted on the 3D HCT-116 model better reproducing the cancer environment. The anti-proliferative effects of Et_3_PAuCl and nano-Et_3_PAuCl in CRC cells are likely due to a dual effect on apoptosis and autophagy. Also, of interest, is the significant difference in terms of signaling pathways related to cell survival affected by Et_3_PAuCl or nano-Et_3_PAuCl: indeed, both inhibit efficiently the AKT pathway, while only nano-Et_3_PAuCl is able to inhibit the ERK pathway. The fate of PLGA-PEG nanoparticles could be traced thanks to the presence of an attached fluorophore. We observed that the nanoparticles are progressively internalized in CRC cells over 4 h observation. The time-dependence of the internalization process was also independently confirmed by ICP-AES experiments. In conclusion, our results suggest that nano-Et_3_PAuCl alone elicits cytotoxic effects in CRC cells most likely through inhibition of different signaling pathways, i.e. ERK and AKT, improving the antitumoral activity of the free gold complex Et_3_PAuCl. Indeed, the data we collected on CRC cell line suggest a peculiar efficacy of nano-Et_3_PAuCl on ERK pathway, which is almost totally inhibited.

A previous mechanistic investigation conducted by our research group had revealed that Et_3_PAuCl, analogously to AF, inhibits thioredoxin reductase (TrxR) (Marzo et al. 2017) and it was recently demonstrated that targeting TrxR inhibits the growth of cancer cells by inducing apoptosis through activation of the MAPK signaling pathway and, additionally, by the inhibition of AKT/mTOR pathway that in turn regulates autophagy (Lei et al. 2018; Comfort et al. 2011). In addition, AF has been reported to induce ERK and AKT inactivation in lung cancer cells (Fan et al. 2014).

These recent findings nicely support our preliminary data on nano-Et_3_PAuCl.

## Supplementary Information

Below is the link to the electronic supplementary material.

Supplementary file1 (DOCX 191 kb)
