# Supplementary material for: Anticancer effects against colorectal cancer models of chloro(triethylphosphine)gold(I) encapsulated in PLGA–PEG nanoparticles
Source: Biometals. 2021 Apr 27;34(4):867–79. doi: 10.1007/s10534-021-00313-0 (PMC8313464; doi:10.1007/s10534-021-00313-0)
Supplement: Supplementary file 1 — Supplementary file1 (DOCX 191 kb) [file 10534_2021_313_MOESM1_ESM.docx]

Supporting Information

Anticancer effects against Colorectal Cancer Models of chloro(triethylphosphine)gold(I) Encapsulated in PLGA-PEG Nanoparticles

Alessio Menconi,^[a]^ Tiziano Marzo,^*[b]^ Lara Massai,^[c]^ Alessandro Pratesi,^[d]^ Mirko Severi,^[c]^ Giulia Petroni,^[a]^ Lorenzo Antonuzzo,^[e]^ Luigi Messori,^[c]^ Serena Pillozzi,^*[a,f]#^ Damiano Cirri^*[d]#^

[a] A. Menconi, Dr. G. Petroni, Dr. S. Pillozzi, Department of Experimental and Clinical Medicine, University of Florence, Viale G.B. Morgagni 50, 50134 Firenze, Italy. E-mail: serena.pillozzi@unifi.it

[*b*] Dr. T. Marzo orcid.org/0000-0002-2567-3637, Department of Pharmacy, University of Pisa, Via Bonanno Pisano 6, 56126, Pisa, Italy. E-mail: tiziano.marzo@unipi.it

[*c*] Prof. L. Messori orcid.org/0000-0002-9490-8014, Dr. L. Massai orcid.org/0000-0003-0765-1802, Laboratory of Metals in Medicine (MetMed), Department of Chemistry “U. Schiff”, University of Florence, Via della Lastruccia 3, 50019 Sesto Fiorentino, Italy.

[d] Dr. A. Pratesi orcid.org/0000-0002-9553-9943, Dr. D. Cirri orcid.org/0000-0001-9175-9562, Department of Chemistry and Industrial chemistry (DCCI), University of Pisa, Via G. Moruzzi 13, 56124, Pisa, Italy. E-mail: damiano.cirri@dcci.unipi.it

[e] Dr. L. Antonuzzo, S.C. Oncologia Medica 1, Azienda Ospedaliero-Universitaria Careggi, Florence, Italy.

[f] Dr. S. Pillozzi, DI.V.A.L Toscana S.R.L., Via Madonna del Piano, 6, 50019 Sesto Fiorentino, Italy. E-mail: serena.pillozzi@unifi.it

# These authors equally contributed

Index

[^1^HNMR spectrum 2](#_Toc22823342)

[DLS measurements 2](#_Toc22823343)

[Spectroscopic properties 3](#_Toc22823344)

[Et_3_PAuCl retention 4](#_Toc22823345)

# ^1^HNMR spectrum


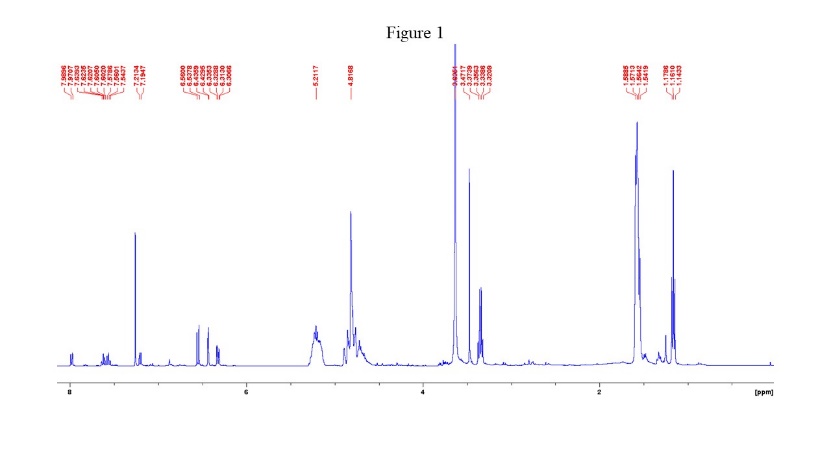


Figure S1. ^1^HNMR spectrum of RhB-C=O-PLGA-C=O-NH-PEG-COOH.

# DLS measurements

| Batch | PDI | Size (nm) |
| --- | --- | --- |
| 1 | 0.17 | 87 |
| 2 | 0.17 | 72 |
| 3 | 0.17 | 71 |
| 4 | 0.22 | 52 |
| 5 | 0.27 | 55 |
| 6 | 0.23 | 74 |
| 7 | 0.18 | 66 |

Table S1. DLS measurements used for determining size and PDI index of nanoparticles.

# Spectroscopic properties


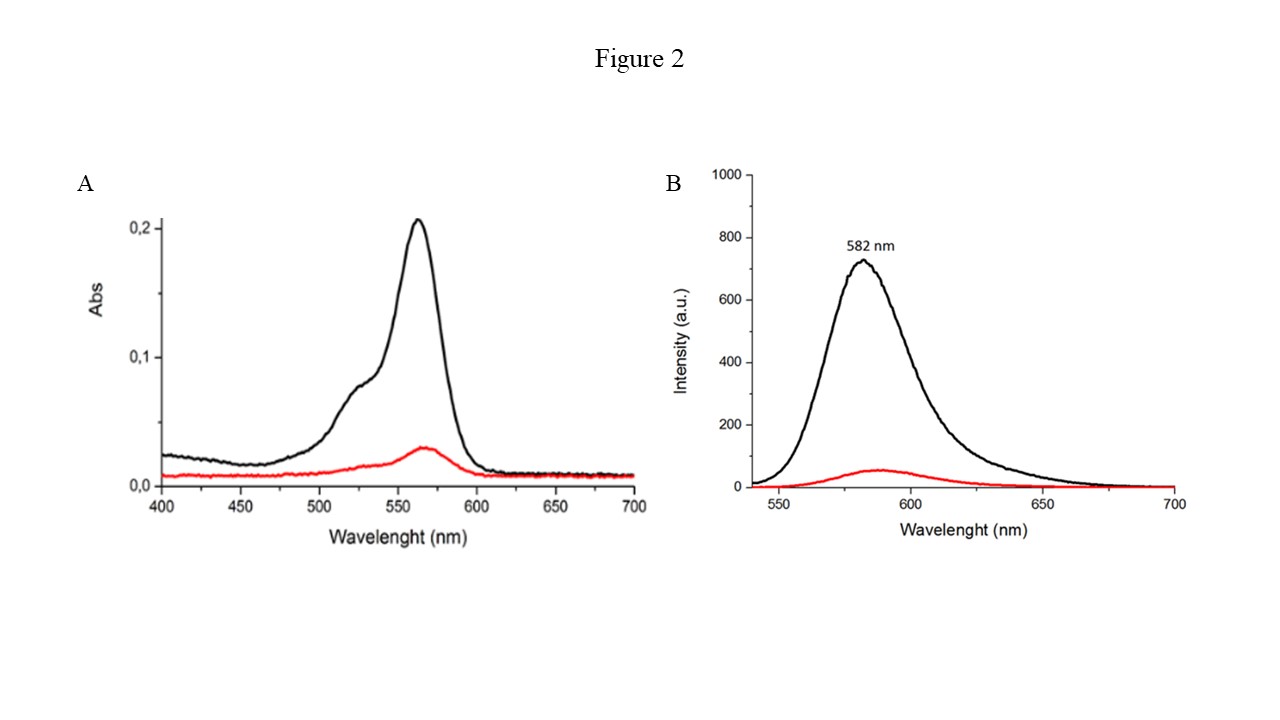


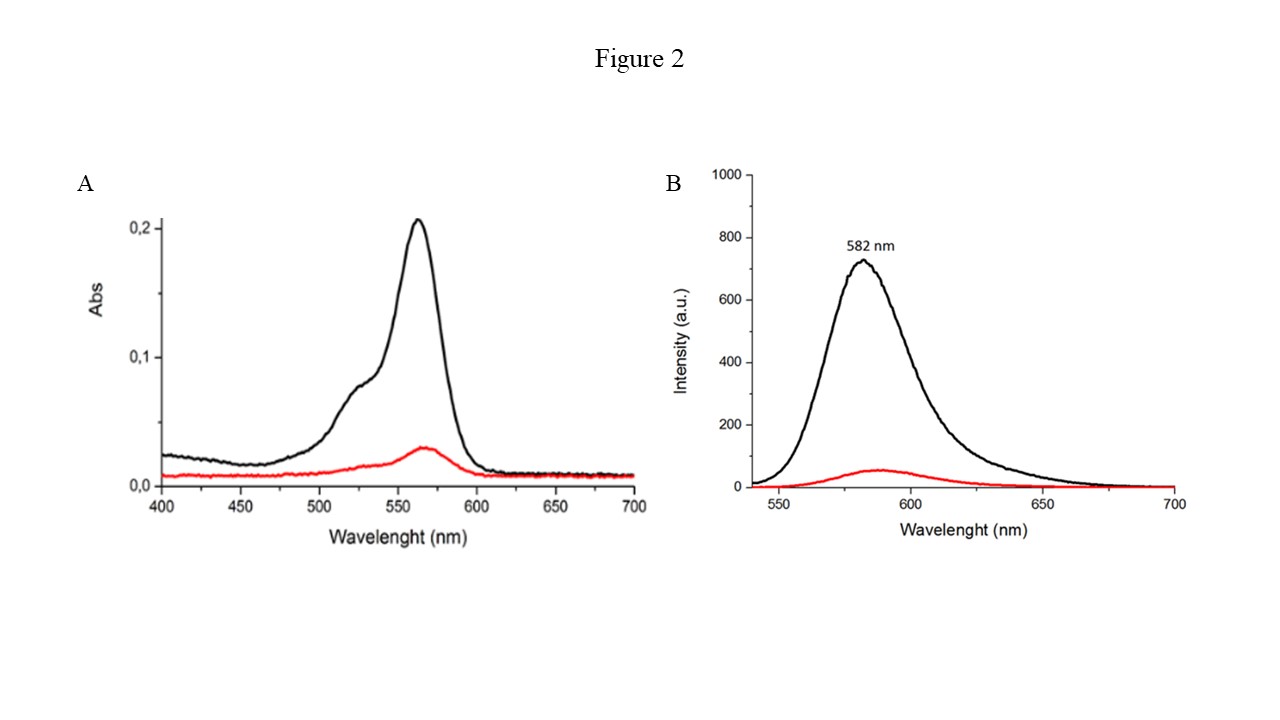


Figure S2. Spectroscopic properties of PLGA-PEG nanoparticles loaded with Et_3_PAuCl. A: Comparison of absorbance profiles recorded on the solution of nanoparticles (black line) and filtered solution (red line). B: Comparison of fluorescence profiles recorded on the solution of nanoparticles (black line) and filtered solution (red line). Spectra were recorded using the same samples of absorbance analysis after an eight-fold dilution with MilliQ water.

# Et_3_PAuCl retention

| Batch | [Gold] (μg/L) | [Released Gold] (μg/L) | Gold retention (%) |
| --- | --- | --- | --- |
| 1 | 7777.3 | 555.4 | 92.9 |
| 2 | 7836.2 | 485.1 | 93.8 |
| 3 | 7982.4 | 694.9 | 91.4 |

Table S2. Determination of the retained amount of Et_3_PAuCl after 24 h at 25° C.


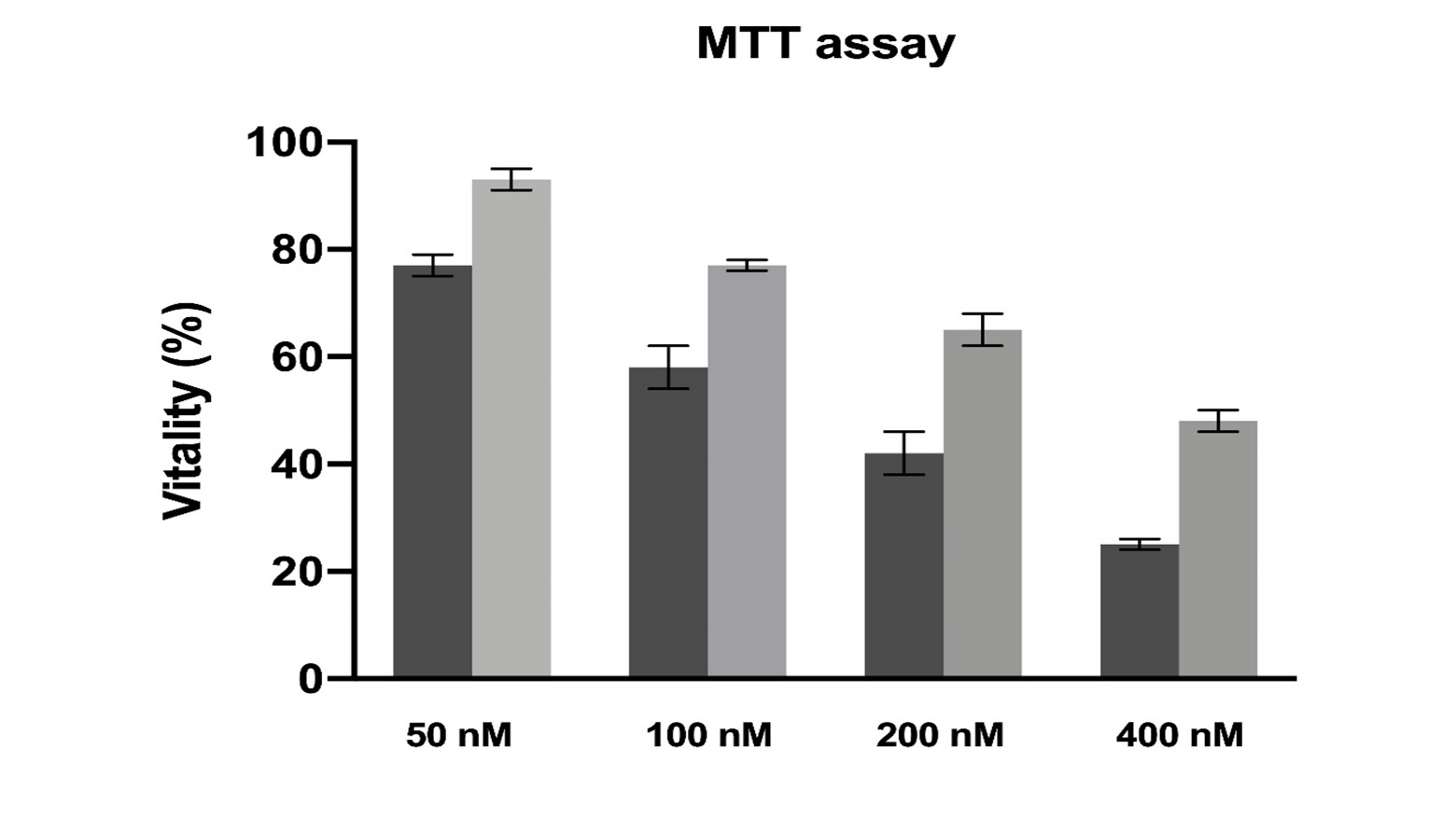


Figure S3. IC_50_ evaluation of Et_3_PAuCl (dark grey) and its encapsulated form (light grey) on HCT-116. Determination performed through MTT assay.
